# From Soil to Salad: Strategies for Reducing Foodborne Illness Outbreaks

**DOI:** 10.1002/fsn3.4521

**Published:** 2024-12-09

**Authors:** Ukti Bimal Sheth, Md Ariful Haque, Min Ji Jang, Samuel Haruna, Tony V. Johnston, Deokyeong Choe, Ying Gao, Seockmo Ku

**Affiliations:** ^1^ Consumer & Industry Service Tennessee Department of Agriculture Nashville Tennessee USA; ^2^ Department of Food Science and Technology Texas A&M University College Station Texas USA; ^3^ School of Agriculture, College of Basic and Applied Sciences Middle Tennessee State University Murfreesboro Tennessee USA; ^4^ School of Food Science and Biotechnology, College of Agriculture and Life Sciences Kyungpook National University Daegu Korea

**Keywords:** agricultural water, foodborne illness, leafy green, pathogen, soil

## Abstract

This study addresses the global issue of foodborne illness, specifically focusing on those resulting from the consumption of leafy green vegetables. It explores the rising trend of consuming minimally processed or raw foods and the imperative of maintaining safety standards starting at the preharvest stage to prevent pathogenic bacterial contamination. The study identifies soil and irrigation water as key sources of pathogens and emphasizes the need for strict preventive measures during production and preharvest. It discusses the challenges of postharvest decontamination and highlights the importance of early‐stage prevention strategies. The paper also examines advanced pathogen detection methods and food safety practices recommended by USDA and FSMA's PSR, including HACCP and LGMA strategies. Aimed at providing insights for consumers and producers, the study underscores the necessity of effective manufacturing strategies to ensure the safety of leafy greens.

## Graphical abstract

This review highlights the importance of early preventive measures against pathogenic bacterial contamination in leafy green vegetables, starting from preharvest stages, due to the increasing consumption of minimally processed or raw foods. It emphasizes the role of soil and irrigation water as contamination sources and advocates for strict production practices and advanced detection methods, in line with USDA and FSMA's PSR recommendations, to ensure food safety.

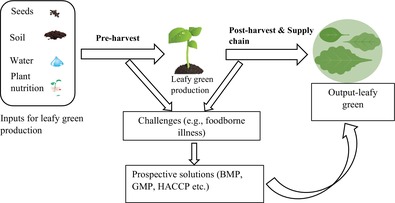

## 1 Introduction

The growing emphasis on health maintenance has shifted consumer preferences toward raw or minimally processed foods versus high‐calorie, processed meals. This trend has driven a significant increase in the consumption of ready‐to‐eat (RTE) leafy greens in wraps, sandwiches, and salads. Reflecting this shift, a recent market analysis revealed that the fresh vegetable market was valued at a significant USD 693 billion in 2023, with an estimated compound annual growth rate (CAGR) of 3.3% from 2024 to 2032 (GMI 2023). Such growth highlights the escalating demand for fresh produce, especially leafy greens rich in vitamins (A, C, E, and K), minerals (iron, magnesium, potassium, and calcium), fiber, and antioxidants, particularly in dark greens like kale, collard, arugula, and spinach (USDA ARS 2013). The 2009 FDA Food Code defines “cut leafy greens” as fresh leafy greens whose leaves have been cut, shredded, sliced, chopped, or torn. The production and processing of various leafy greens, including different lettuce varieties, escarole, endive, spring mix, spinach, kale, arugula, and chard, have surged to meet this rising demand (US FDA 2021a). Statista reported that in the United States, per capita consumption of fresh lettuce (romaine and leaf) rose from 8.4 pounds in 2000 to 15 pounds in 2017 (US Statista 2019). However, in parallel with increasing leafy green consumption, foodborne illness outbreaks associated with those products have also increased (Herman, Hall, and Gould 2015). Factors contributing to this include variations in individual consumption, increased animal breeding near vegetable cultivation sites, irrigation on land previously used for livestock production, and a rise in the number of individuals with health conditions consuming these products (Iwu and Okoh 2019). Maintaining the microbial quality and wholesomeness of greens from farm to table is critical to preventing foodborne illnesses. Once pathogens contaminate leafy greens, eliminating them becomes challenging, so contamination prevention at the preharvest/production stage is essential. This prevention can be achieved by enhancing and monitoring the quality of water, soil, surrounding vegetation, and insecticides used in cultivation (Machado‐Moreira et al. 2019). Other factors, such as farmer hygiene and cross‐contamination risks, are also significant (US FDA 2021b). The raw consumption of these greens in ready‐to‐eat (RTE) forms necessitates stringent measures to safeguard against potential hazards. Effective pathogen prevention at the preharvest stage is vital, where greens are exposed to potential contaminants in irrigation water, soil, compost, manure, fertilizers, seeds, equipment, and human contact. While fresh produce is cultivated in open fields prone to innumerable contamination sources, most pathogen transmission occurs through irrigation water, adjacent cattle, and soil (Iwu and Okoh 2019). Addressing these preharvest challenges is possible through the adoption of food safety practices outlined by the United States Department of Agriculture (USDA). These include USDA's Good Agricultural Practices (GAP), the Food Safety Modernization Act's Produce Safety Rules (PSR) (US FDA FSMA 2023a, 2023b), Hazard Analysis Critical Control Point (HACCP) plans, and the Leafy Green Marketing Agreement (LGMA), which offer preventative approaches from preharvest to postprocessing (Beecher 2013a, 2013b). The objective of this paper is to illustrate the challenges and offer solutions in the production of edible leafy greens. By identifying these challenges and offering solutions at both the pre‐ and postharvest stages, this research review aims to benefit consumers by mitigating health hazards and encourage growers to adopt production practices that minimize foodborne pathogen risk.

## 2 Leafy Green Foodborne Illness Outbreaks

The US Centers for Disease Control and Prevention (CDC 2023a, 2023b) reports that approximately one in six Americans suffers from a foodborne illness annually. Common pathogens linked to fresh produce include bacteria, viruses, fungi, and parasites. Among these, the typical pathogens infecting leafy greens are *Escherichia coli* (0157:H7), *Salmonella* spp., *Listeria monocytogenes*, *Shigella* spp., *Cyclospora*, and Norovirus (Alegbeleye, Singleton, and Sant'Ana 2018). From 1998 to 2008, most foodborne illness outbreaks were associated with leafy vegetables alone, more than any other food category. Between 2014 and 2021, 78 foodborne illness outbreaks were linked to leafy greens, primarily lettuce (CDC 2023a, 2023b). In the United States, three significant multistate foodborne outbreaks involving leafy greens occurred in 2019 and 2020, resulting in 918 illnesses and five deaths (CDC). From 1980 to 2016, around 571 food outbreaks in the United States were reported, leading to 72,855 infections and 173 fatalities. Of these outbreaks, 51.7% were related to fresh leafy greens. Fresh leafy greens become a public safety hazard when the quality of the soil and irrigation water used for their cultivation deteriorates. One of the largest multistate *E. coli* outbreaks in 2006, which led to 210 illnesses across 36 states, 96 hospitalizations, and five deaths, was traced back to contaminated irrigation water at a farm in Yuma County, Arizona (US FDA 2019). Another major multistate (27 states) *E. coli* 0157:H7 outbreak, linked to romaine lettuce, infected 167 and hospitalized 85, was traced to agricultural water and soil in California's Salinas Valley (CDC 2019). Table 1 lists many of these outbreaks and produce recalls linked to fresh produce and includes the source, associated pathogen, and affected population.

**TABLE 1:** United States food illness outbreaks from 1995 to 2023 caused by pathogenic strains sourced from soil, water, and poor hygienic practices infecting green leafy produce. The data listed are retrieved primarily from CDC, Food Safety News, and other sources.

Year	Causative pathogen	Fresh produce (linked)	Illness reported	Epidemiological (traced) source
June 2023	*Listeria monocytogenes*	Leafy greens	19	Unknown
April 2022	*Listeria monocytogenes*	Packaged salad	18	Unknown
March 2022	*Listeria monocytogenes*	Prepackaged salad	10	Unknown
January 2022	*E. coli* O157:H7	Packaged salad	10	Unknown
December 2021 (CDC 2022)	*E. coli* O157:H7	Baby spinach	14	Processing facility
October 2021 (CDC 2021)	*Salmonella typhimurium*	Prepackaged salad	31	Unknown
December 2020 (CDC 2020a, 2020b, 2020c)	*E. coli* O157:H7	Leafy greens	40	Farms
September 2020 (CDC 2020a, 2020b, 2020c)	*Cyclospora*	(bagged) Salad mix	701	Processing facility
September 2019 (CDC 2020a, 2020b, 2020c)	*E. coli O157:H7*	Romaine lettuce	167	Farm/water
November–December 2017 (CDC 2017)	*STEC O157:H7*	Romaine lettuce	17	Unknown
March 2016 (CDC 2016a, 2016b, 2016c)	*Listeria monocytogenes*	(packaged) Salad	19	Improper handling in the processing plant
January 2016 (CDC 2016a, 2016b, 2016c)	*Listeria monocytogenes*	Frozen vegetables	9	Improper hygiene during processing
November 2013 (CDC 2013)	*STEC O157:H7*	Ready to eat (RTE) salad	33	Supply chain
October 2011 (CDC 2012)	*E. coli O157:H7*	Romaine lettuce	58	Unknown
May 2010 (CDC 2010)	*E. coli 0145*	Shredded romaine lettuce	26	Unknown
July 2009 (CDC 2009)	*Salmonella serotype saintpaul*	Raw alfalfa sprout	235	Improper processing at the facility
Sept 2006 (CDC 2006)	*E. coli O157:H7*	(Triple‐washed bagged) Spinach	199	Unknown but suspected animal feces nearby harvest
September 2003 (Flynn 2009)	*E. coli O157:H7*	(Prewashed) lettuce	40	Wash water quality
February 1999 (Flynn 2009)	*E. coli O157:H7*	Iceberg lettuce	72	Restaurant/ improper hygiene
May 1996 (Flynn 2009)	*E. coli O157:H7*	Mesclun lettuce	61	Poor quality of irrigation water
July 1995 (Flynn 2009)	*E. coli O157:H7*	Leaf lettuce	92	Animal's presence near water (used for lettuce)

Rapid detection methods for pathogens are increasingly replacing traditional, time‐consuming, and labor‐intensive methods. These rapid techniques are not only consistent and reliable but also provide accurate and timely results. They are categorized into three main types: (i) Nucleic acid‐based methods, including simple polymerase chain reaction (PCR), multiplex PCR, real‐time PCR, nucleic acid sequence‐based amplification (NASBA), loop‐mediated isothermal amplification (LAMP), and oligonucleotide DNA microarray methods; (ii) biosensor‐based methods, encompassing mass‐based, optical, and electrochemical biosensors; and (iii) immunology‐based methods, such as enzyme‐linked immunosorbent assays (ELISAs) and lateral flow immunoassays. Among these, PCR and other nucleic acid techniques are highly sensitive and specific, providing the most accurate results quickly; hence, they are commonly used to identify the causative agent(s) in foodborne illness outbreaks (Law et al. 2015).

### 2.1 Potential Pathogen Sources and Their Challenges

**FIGURE 1:**
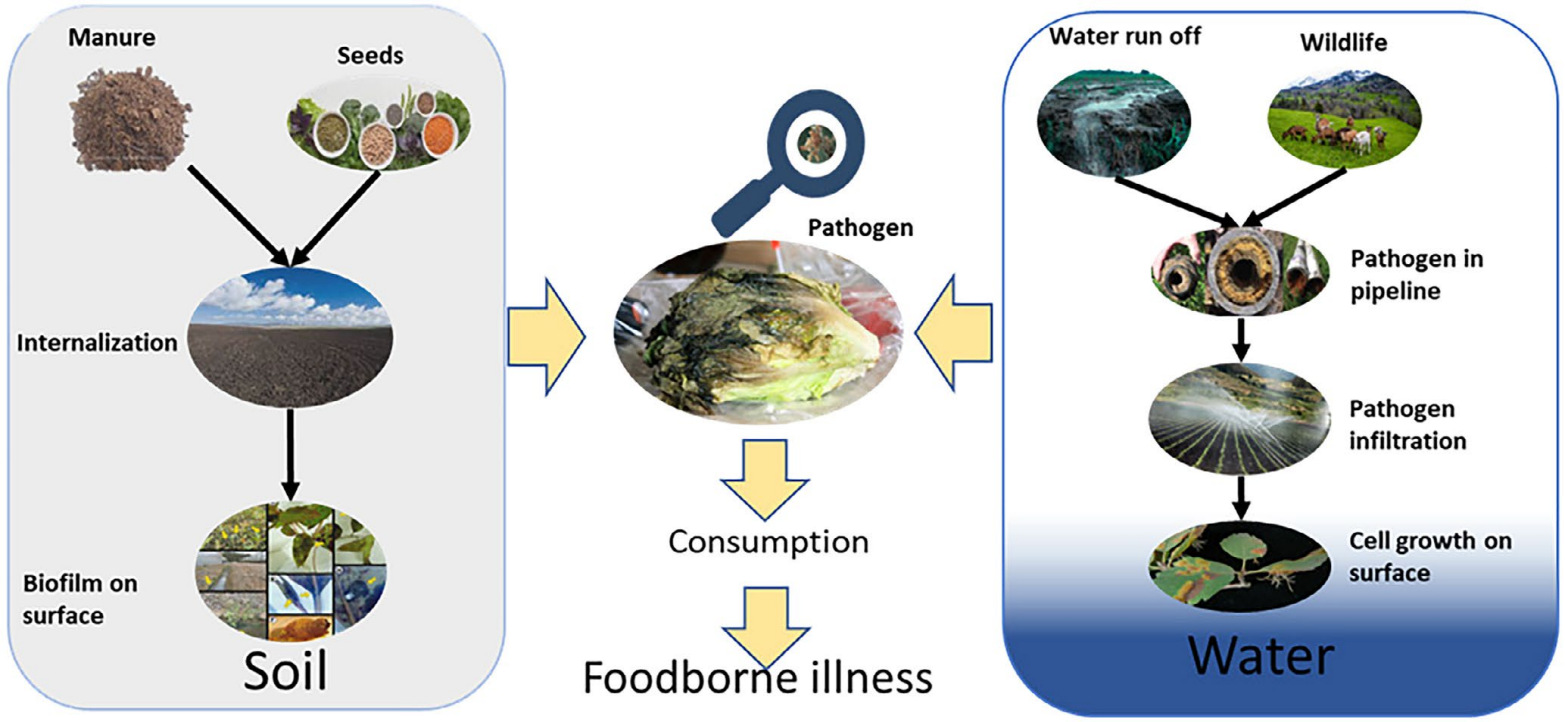
Potential preharvest pathways for the entry of pathogens into leafy greens from soil and water. Other subfactors, such as manure, seeds, surface water, and livestock grazing, can ruin the quality of soil and water used for the cultivation of leafy greens (Modified from Alegbeleye, Singleton, and Sant'Ana (2018)).

Fresh produce is cultivated on farms exposed to numerous known and unknown factors that impact microbiological quality. These factors vary depending on the location, environment, landscape, previous land use, and proximity to animal production and breeding facilities. Surrounding factors can promote pathogen spread and recurrence through vehicles like contaminated soil, water, improperly treated manure/compost, and nearby cattle activities (Alegbeleye, Singleton, and Sant'Ana 2018; Gil et al. 2015). When these vehicles contact leafy greens, pathogens persist and internalize, compromising produce quality and creating a public health hazard. Figure 1 illustrates the potential pathways and sources of pathogen infiltration in leafy greens (Alegbeleye, Singleton, and Sant'Ana 2018).

### 2.2 Sources of Soil Contamination

Soil, comprising minerals, organic matter, living organisms, gases, and water, is primarily categorized into clay, sand, and silt (Needelman 2013). It hosts a variety of microorganisms, including bacteria, fungi, algae, protozoa, nematodes, and actinomycetes, which aid in decomposing soil and organic matter (Kannojia, Sharma, and Sharma 2019). Viruses, although not naturally present in soil, can grow in it if introduced through sources like sewage systems (Brevik and Burgess 2014). Human or animal fecal matter in soil can release human or enteric pathogens, leading to gastrointestinal infections and illnesses through the fecal–oral route. Common pathogens include bacteria, viruses, and protozoa (Santamaría and Toranzos 2003).

#### 2.2.1 Soil Amendments or Manure

Animal breeding or nurturing activities on small and large farms, often adjacent to crop production areas, use animal manure as a soil amendment and fertilizer to enhance soil fertility. Manure, a biodegradable organic mass, should be spread as composted manure after being stored to kill pathogens and dried for even spreading (US EPA 2023). Raw manure, which can contain pathogenic strains, can transmit pathogens to leafy produce if used improperly or in noncomposted form. Studies suggest that cow dung fertilizer increases the survival of enteric pathogens. Manure amendments, whether solid or liquid, provide environments conducive to pathogen persistence, with survival rates varying based on the manure application extent, soil aeration, pH, and moisture content. The USDA National Organic Program Regulation mandates a 120‐day gap between applying raw or untreated manure and harvesting fresh produce like leafy greens and a minimum of 90 days for produce not contacting the soil (Iwu and Okoh 2019). *Escherichia coli* and *Salmonella* spp. are primarily sourced from cattle manure, as their diets create ideal conditions for pathogen survival (Alegbeleye, Singleton, and Sant'Ana 2018). The ideal carbon‐to‐nitrogen ratio in composted manure should be 30:1 (Browning 2013).

#### 2.2.2 Animal Activities

Contamination in leafy greens leading to foodborne illness can be mediated by eliminating animals pastured or raised near cultivation areas. Animal intrusion, whether deliberate or accidental, causes pathogen colonization of greens through direct contact or cross‐contamination. Animals can shed zoonotic pathogens like *Campylobacter* spp. and *E. coli* (0157: H7) (Iwu and Okoh 2019). Limiting contact with domestic animals is possible with physical barriers like fences, but controlling wild animals is more challenging. An *E. coli* 0157:H7 infection in California baby spinach, which affected 200 individuals and caused three deaths, was linked to animal activities near irrigation water, although the exact transmission pathway remains unconfirmed (Beecher 2013a, 2013b). Grazing areas should be downhill from cultivation fields to prevent runoff contamination.

#### 2.2.3 Human Activities

Anyone entering a farm, be it workers, visitors, or customers, can be a carrier of human pathogens. Lack of hygiene, such as inadequate handwashing or unclean clothing/footwear, can lead to leafy produce contamination. Sick farm workers or those with open wounds can shed harmful microorganisms onto produce (Beecher 2013a, 2013b). The FDA reports that *Cyclospora cayetanesis*, a human parasite, can infect farm workers, leading to cyclosporiasis, a contagious illness causing diarrhea (US FDA 2022). Inadequate handwashing post‐infection can spread pathogens in fresh vegetables (US FDA 2022).

### 2.3 Agricultural Water

The CDC defines agricultural water as water used in cultivating crops (irrigation) and in animal management practices. This includes water for irrigation, pesticide and fertilizer applications, reducing crop temperatures, and other farm‐related activities. Agricultural water sources encompass groundwater (wells), rainwater (cisterns and barrels), and surface water (ponds, rivers, streams, and canals). Some farmers also rely on municipal or rural water systems for irrigation and livestock needs (CDC 2016a, 2016b). The United States Geological Survey (USGS) reports that approximately 56% of surface water and 44% of groundwater were utilized daily for agriculture between 1950 and 2015, emphasizing the significance of surface water in US agricultural production (Figure 1). Such water sources can potentially harbor pathogens like Norovirus, *Shigella* spp., *Cyclospora*, verotoxin‐producing *E. coli*, *Cryptosporidium*, and *Yersinia* spp. (Uyttendaele et al. 2015).

#### 2.3.1 Irrigation Water

**FIGURE 2:**
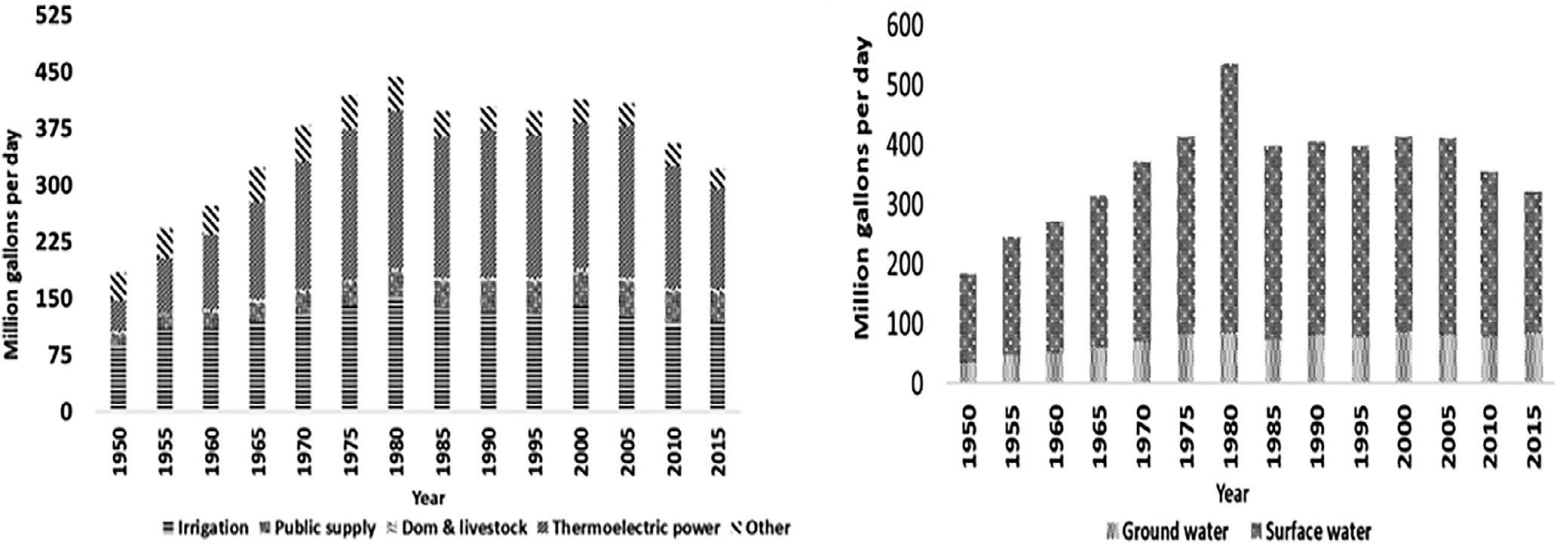
Daily average consumption of ground and surface water used as irrigation water in the USA from 1950 to 2015 (modified from Uyttendaele et al. (2015)).

Water is vital for the growth and harvesting of leafy greens and is often supplied through irrigation systems, particularly during dry seasons. Figure 2 depicts the estimated irrigation water withdrawals from 1950 to 2015, highlighting the extensive use of surface and groundwater for irrigation (Uyttendaele et al. 2015). Previous research suggests that many foodborne illness outbreaks are linked to poor irrigation water quality, especially where water scarcity leads farmers to use minimally treated wastewater for preharvest or harvest irrigation (Alegbeleye, Singleton, and Sant'Ana 2018; Gil et al. 2015). The quality of irrigation water, including natural sources like surface water, has deteriorated due to urban expansion, climate change, and industrial development. Agricultural water quality is assessed by the presence of enteric pathogens, and pathogen survival is influenced by factors like the water source, seasonal impacts, temperature, and irrigation methods (drip or surface irrigation) (Matthews 2013). Studies have indicated that SARS‐CoV‐2 might be considered an enteric pathogen, potentially impacting the intestinal tract via recycled wastewater irrigation of leafy greens (Oliver et al. 2020; Gu, Han, and Wang 2020; Lodder and de Roda Husman 2020). The duration of water contact is crucial, particularly with water containing a high pathogenic load. For instance, using contaminated irrigation water close to harvest time significantly increases health risks to consumers. Hence, maintaining an adequate interval between irrigation and harvest is essential (Alegbeleye, Singleton, and Sant'Ana 2018).

#### 2.3.2 Sources of Irrigation Water

Effluent water, often used multiple times by small farmers, has various advantages including nutrient enrichment, lower cost, and availability. However, its quality can be compromised by organic matter, suspended solids, and microbial contaminants. Consequently, produce irrigated with effluent water requires proper treatment and testing before consumption to prevent public health hazards. Surface water, being open and readily available from rivers, ponds, streams, canals, and dams, is highly susceptible to contamination from livestock, industrial waste, and runoff from contaminated soil. Groundwater, typically safer and more reliable, is stored in aquifers and is useful during water scarcity or droughts. It is recommended for irrigating crops due to its lower pathogen content compared to surface, rain, or effluent water. However, climate factors can significantly affect the quality and quantity of stored water. Rainwater, influenced by the nature of storage containers and vulnerable to contamination from birds, insects, and other small animals, is another accessible source when water demand spikes (Iwu and Okoh 2019).

#### 2.3.3 Methods of Application

Irrigation methods include surface (furrow/flood), drip/trickle, sprinkler, and subsurface delivery. The drip/trickle method is relatively safe for leafy greens, provided the water is pathogen‐free and contacts only the plant's roots. In contrast, the overhead sprinkler method poses risks, as the edible parts of the produce are exposed to water. Surface irrigation methods like furrow and flood are less risky since water flows along the soil, minimizing exposure to the harvested portions of the plants. Timing is also crucial; a longer gap between irrigation and harvest reduces the risk of pathogen invasion, as few pathogens can survive post‐irrigation environmental conditions on the plants (Mahmoud 2018).

#### 2.3.4 Persistence of Pathogens in Soil and Water

While no research on COVID‐19 outbreaks from leafy green produce has been identified, SARS‐CoV‐2 poses a potential public health threat. Its stability in aqueous environments—up to 4 days in wastewater and several weeks in tap water—could lead to outbreaks if recycled wastewater is applied to leafy greens before harvest (Florek et al. 2014). The survival of pathogens depends on physical, chemical, and biological factors. For example, *Listeria monocytogenes* can endure a wide range of environmental conditions, while *E. coli* 0157:H7 and *Salmonella* survive only under specific conditions of temperature, humidity, and sunlight (US FDA 2016). Pathogen introduction, presence, and colonization depend on soil type, pH, organic matter, moisture content, biotic interactions, composition, temperature, and farming practices. *Escherichia coli* and *Salmonella* can persist in soil for 7–25 weeks, depending on soil temperature and humidity (Beecher 2011). Pathogen proliferation is greater in amended (manured) soil than in unamended soil. Studies have shown that *Salmonella typhimurium* survives better in high‐pH clay soil than in silty or loamy soil. The application of cow manure to land can sustain *E. coli* survival for about 6 weeks (Iwu and Okoh 2019). Reduction in soil moisture also decreases viral and bacterial numbers, with clay acting as a barrier by adsorbing microorganisms to its surface, thereby offering protection against parasites. The presence of organic matter, high carbon content, and low temperatures can foster the growth of pathogenic bacteria and viruses.

## 3 Pathogen Defense/Antibiotic Resistance (AR)

In the absence of heat treatment, fresh greens are often treated with disinfectants like chlorine to mitigate waterborne disease outbreaks. However, this practice has inadvertently promoted the growth of antibiotic‐resistant bacteria (ARB) and the horizontal transfer of antibiotic resistance genes (ARG), both intracellular and extracellular. This development poses a significant public health hazard (Jin et al. 2020). When leafy greens are contaminated with pathogens at the preharvest or postharvest stages, serious, sometimes life‐threatening, health complications can arise, particularly in immunocompromised individuals. If these pathogens are antibiotic‐resistant, the effectiveness of standard treatments diminishes, prolonging the healing process (Iwu and Okoh 2019). Notable ARB pathogens include *Salmonella*, *Streptococcus aureus*, *Campylobacter*, *Yersinia* spp., and *Staphylococcus pneumonia* (Hashempour‐Baltork et al. 2019).

### 3.1 Methods for Killing Pathogens PostHarvest and Before Processing

It is crucial to adopt measures that prevent pathogen persistence and proliferation in the field before the produce reaches the processing or packaging facility. Most pathogenic strains are eliminated by heat processing, but since leafy greens are often consumed raw, alternative methods are necessary. Irradiation, chemical disinfectants, pulsed light, and pulsed electric fields can reduce pathogen loads but offer significant disadvantages. Although washing with water removes debris and organic matter, it risks cross‐contamination from the other produce. Sanitizing solutions containing chlorine, peroxyacetic acid (PAA), ozone, organic acids, electrolyzed water, and other organic acids have been proven effective against pathogens (Pahariya, Fisher, and Choudhary 2022). A review by Mahajan et al. (2014) showed that PAA reduced L. monocytogenes and *E. coli* 0157:H7 on lettuce and achieved a 5‐log reduction of *Enterobacter sakazakii*.

#### 3.1.1 Application of Pesticides

Pesticides, crucial for controlling weeds and pests, are widely used by farmers to enhance yields and reduce vegetable losses. Approximately 33.33% of agricultural products are produced using pesticides, and their absence could lead to significant production losses in fruits, vegetables, and cereals. However, overuse of pesticides can negatively impact birds, animals, and aquatic life through direct and indirect consumption. Pesticidal residues in produce can also be harmful to human health (Tudi et al. 2021). Macieira, Barbosa, and Teixeira (2021) reported that the annual consumption of agricultural pesticides is up to 2.5 million tons, with raw or minimally processed produce often containing high levels of insecticides and fungicides. Additionally, the presence of heavy metals in nature, such as mercury, cadmium, lead, arsenic, etc., can deplete the body's nutrient levels and cause severe harm, including kidney failure, genetic diseases, and immunological disorders.

#### 3.1.2 Use of Chemical Mechanisms in the Control of Pathogens on Leafy Greens

Using water alone to clean fresh produce can result in cross‐contamination. The irregular surface structure and natural openings (stomata) of plants enable bacteria to adhere to the leaves even after washing. Biofilms produced by microorganisms further support adhesion. Sanitizers are effective against pathogens like *Salmonella* and *Escherichia coli O157:H7*, and multiple physical and chemical disinfection methods are used to reduce microbial loads on fresh produce (Deng et al. 2020). Combining disinfectants is considered an approach that may eliminate pathogens from fresh produce. Disinfection is a necessary step before packaging or consuming leafy greens. Based on studies on the use of organic acids, chlorine, ozone, electrolyzed oxidizing (EO) water, hydrogen peroxide, sodium chloride, sodium sulfite, sodium hypophosphite, and quaternary ammonium compounds to reduce microbial loads on fresh produce, microbial diversity and composition are perhaps the most important factors in sanitizer selection.

#### 3.1.3 Chlorine and Chlorine Compounds

Chlorine dioxide (ClO_2_) is an effective sanitizer based on its strong oxidizing properties (1.57 V). It has been shown to inactivate both Gram‐positive and Gram‐negative bacteria at 0.1 ppm with a minimal contact time and without producing carcinogenic by‐products like trihalomethanes (Praeger, Herppich, and Hassenberg 2018). Its antibacterial mechanism involves reacting with vital amino acids and disulfide bonds, disrupting cell metabolism and RNA, destabilizing cell membranes, altering membrane permeability, and interrupting protein synthesis. Park and Malka (2022) found that gaseous ClO_2_ achieved a 4.07‐log reduction in bacteria, proving more effective than other sanitizers. The CDC indicates that in the absence of organic matter, free available chlorine forms such as HOCl, OCl–, and Cl_2_ are effective in killing mycoplasma at a concentration of 25 ppm and can eliminate vegetative bacteria at concentrations lower than 5 ppm, achieving this biocidal effect within seconds (CDC 2008). However, concentrations over 250 ppm can alter the sensory properties of some leafy greens (CDC 2008). Although explosive at high concentrations and temperatures, chlorine dioxide remains effective against most microbes at concentrations of 3–5 ppm in clean water.

#### 3.1.4 Organic Acids

Research has shown that organic acids are biocidal to microorganisms, acting by damaging the function of the cell wall. The undissociated form of organic acid diffuses through the microbial membrane, inhibiting enzymatic reactions and modifying proteins and the DNA structure (Lepaus, Rocha, and São José 2020). Most organic acids exhibit antibacterial activity by reducing environmental and cellular pH and increasing anion accumulation, and they are recognized as GRAS by the FDA (Wang et al. 2019). Different organic acids have varying mechanisms of action on bacterial cells; however, common antimicrobial mechanisms include reducing environmental pH, increasing anions, and causing cell enlargement that ruptures the cytoplasmic membrane. Citric acid, for instance, acts by binding with metal ions and disrupting bacterial homeostasis. In a study by Wang et al. (2019) lactic acid was found to be the most effective sanitizing agent among other acids. Kundukad et al. (2020) reported that lactic acid at pH 3 showed no detectable colonies in rocket leaves. While recognized as GRAS, the effectiveness of organic acids against microorganisms is generally low and requires high concentrations and long exposures.

#### 3.1.5 Electrolyzed Oxidizing (EO) Water and Salt Solution

Electrolyzed oxidizing water, produced by the electrolysis of dilute sodium, magnesium, or potassium chloride solutions, is considered valuable for maintaining food safety standards as it has antimicrobial properties. EO water techniques are used postharvest on produce to kill bacteria and extend the shelf life. High concentrations or prolonged application of EO water significantly reduce bacterial loads without adversely affecting lettuce and spinach leaves. The bactericidal activities of EO water are attributed to its low pH, high oxidation–reduction potential (ORP), and active chlorine content. Iram, Wang, and Demirci (2021) suggested that the low pH of acidic EO water increases the susceptibility of cells to active chlorine, leading to cell destruction. An effective concentration of 20 mg/L of free chlorine is recommended for complete pathogen removal. Park, Hung, and Chung (2004) found that Salmonella cells were effectively inactivated with 1.0 mg/L EO water with residual chlorine, and inactivation levels increased with concentration up to 5.0 mg/L.

#### 3.1.6 Hydrogen Peroxide

Hydrogen peroxide is effective against a wide range of microorganisms, including bacteria, yeast, fungi, viruses, and spores, with an oxidative potential of 1.8 V. It is recognized as GRAS by the US FDA and EPA and is environmentally friendly as it breaks down into oxygen and water (US FDA 2023a, 2023b; US EPA 2024). Hydrogen peroxide acts on bacteria by producing hydroxyl‐free radicals that attack membrane lipids, DNA, and other essential cell components. The FDA approves hydrogen peroxide as a sanitizing solution on fresh produce at levels not exceeding 59 ppm water (US FDA 2023a, 2023b). Its use in combination with acetic acid, peroxyacetic acid (PAA), has become increasingly common.

#### 3.1.7 Ozone (O_3_ ) in Vegetable Industries

Ozone, recognized as GRAS in 1995 in the USA for disinfecting bottled water, gained GRAS status for direct contact with foods in 1997 and was approved by the FDA in June 2001 for use in gas and aqueous phases as an antimicrobial additive. Ozone's high oxidative potential (2.1 V) stems from its ability to donate an oxygen atom and react with other compounds. It destroys microorganisms through the progressive oxidation of vital cell components. Sarron, Gadonna‐Widehem, and Aussenac (2021) reported that dipping fresh‐cut iceberg lettuce in ozonated water at 4 mg/L for 2 min effectively reduced mesophilic, psychotropic bacteria and *Enterobacteriaceae* by 1.3‐ to 1.7‐log CFU/g. Factors influencing ozone's efficacy as a sanitizer include pH, temperature, pressure, organic matter, relative humidity, microbial strain characteristics, physical state of bacteria, natural microflora, and cell population size. Ozone is typically used in concentrations ranging from 0.5 to 2 ppm, but higher concentrations above 4 ppm can be corrosive and harmful to humans over prolonged exposure. Table 2 shows the summary on the use of chemicals to inactivate pathogens in the food industry.

**TABLE 2:** Chemicals’ usage for pathogen inactivation (Information contained in this table was adopted and modified from Joshi et al. (2013); Hopkins et al. 2021).

Chemical/sanitizer	Recommended concentration levels	Advantages	Disadvantages	Bacteria log reduction
Chlorine	300 mg/L	Cheap, easy to use. Effective Can be bought over the counter	Can be corrosive to equipment It degrades with age and exposure to sunlight and heat Does not work with high organic loads in water It is pH‐dependent	1.7
Chlorine dioxide	100 ppm	Less reactive than chlorine with organic load in water Effective at a wide range of pH	Breaks down with exposure to sunlight. Has to be generated on‐site before use Cost about twice as much as chlorine	3.08
Organic acids Acetic acid Lactic acid PAA	2% 2% < 50 ppm	Noncorrosive to equipment Works at a wide range of pH values and temperatures Not as sensitive to organic load as chlorine	Cost more than that of chlorine Unpleasant odor may occur Loses its effectiveness in the presence of metal ions	3 3 2.5
Electrolyzed water	250 ppm	Safe and free of toxicity Can be easily produced on‐site and is less expensive	It has a very short shelf life	4.8
Hydrogen peroxide	59 ppm	Environmentally friendly It is declared Generally Recognized as Safe by FDA	Higher concentrations can alter the sensory properties of some vegetables Unstable, degrades fast	2.9
Ozone gas	5.2 mg/L			1.6
Acidified sodium chlorite	500–1200 ppm at pH 2.3–2.9	Better at killing microorganisms than chlorine because of low pH	Generated on‐site by blending before use	

### 3.2 Pathogen Internalization and Biofilms

Internalization refers to the migration of microbes from the external environment into the internal tissues of plants or produce surfaces (Riggio, Jones, and Gibson 2019). Pathogens infiltrate leafy produce, seeking protection from adverse environmental conditions to survive and proliferate, thus infecting the edible parts of leafy greens. Plants possess natural defense mechanisms, such as waxy cuticles and smooth skin, that help inhibit pathogen entry (Hoagland et al. 2018). Leafy greens are particularly delicate and susceptible to damage from both biotic (plants, animals, bacteria, and trees) and abiotic (water, air, sunlight, and temperature) stress factors. Pathogens can enter produce, such as lettuce, through tissue structures like stomata or lenticels, roots, or large openings created by abrasions, wounds, and cuts (Alegbeleye, Singleton, and Sant'Ana 2018). *Escherichia coli 0157:H7* persisted on spinach leaves after spray irrigation, but not on lettuce plants, due to a plant–microbe relationship (Erickson et al. 2014). Another study found that Salmonella affixation was greater on romaine lettuce than on iceberg lettuce. Following invasion, microbial cells attach to one another and to plant tissues, roots, and some intercellular passages in the leaves with a sticky extracellular polymeric substance (EPS), forming biofilms. These biofilms enhance bacterial defenses against adverse conditions like ultraviolet radiation, nutrient deficiency, plant defense systems, and disinfecting chemicals (Hoagland et al. 2018). Bacteria can also colonize and form biofilms on irrigation pipes, contaminating water and subsequently the plant or soil (Pachepsky et al. 2012). This issue can be mitigated by flushing pipes before use and monitoring the water quality.

## 4 Regulations to Prevent Contamination of Leafy Greens

### 4.1 Food Safety Modernizing Act (FSMA)

In response to increasing foodborne illness outbreaks from leafy greens and to protect public health, particularly among vulnerable populations, the Food Safety Modernization Act (FSMA) was signed into law on January 4, 2011. This act focuses on preventing food contamination before it occurs rather than addressing it post‐occurrence (Hoagland et al. 2018). FSMA's “Produce Safety Rule” (PSR), implemented in 2016, established minimum standards for the safe growing, harvesting, packing, and holding of fresh fruits and vegetables (US FDA FSMA 2023a, 2023b). Farms covered under PSR must adhere to standards that limit bacterial growth on produce and reduce associated illnesses. These standards include agricultural water quality, employee health, soil amendments, proximity to cattle, and the use of buildings, tools, and equipment (US FDA 2018).

### 4.2 Good Agricultural Practices (GAPs)

Good Agricultural Practices (GAPs) were developed in response to FSMA to address the potential of food outbreaks starting at the farm level. GAPs assist farm growers/producers by identifying the potential sources of contamination and suggesting remediation methods, focusing on handler hygiene, surface, water, and soil contamination (Vincent, Khouryieh, and Stone 2015). GAP rules guide farmers in adopting measures to prevent pathogen transmission. States like Arizona and California have implemented their own audit programs, such as the Leafy Green Marketing Agreement (LGMA), to address specific foodborne illness outbreaks in leafy greens (USDA AMS 2023).

### 4.3 Hazard Analysis and Critical Control Point (HACCP)

Hazard Analysis and Critical Control Point (HACCP) is overseen by two US government agencies: the US Food and Drug Administration (US FDA) and the United States Department of Agriculture (USDA). The USDA regulates HACCP specifically for meat and poultry, while the US FDA oversees HACCP plans for food and beverages, including leafy greens. The primary goal of HACCP is to mitigate biological, chemical, and physical hazards to an acceptable level by identifying potential risks during food processing (hazard analysis) and implementing controls at specific points where hazards can be eliminated (critical control points) (US FDA 2022). Each food‐producing facility or company must establish and tailor its HACCP system to the product, manufacturing process, and supply chain conditions (US FDA 2022). Moreover, it is crucial to identify hazards based on consumption methods, as different critical control points are required for ready‐to‐eat (RTE) leafy greens and salads intended for washing before consumption.

**FIGURE 3:**
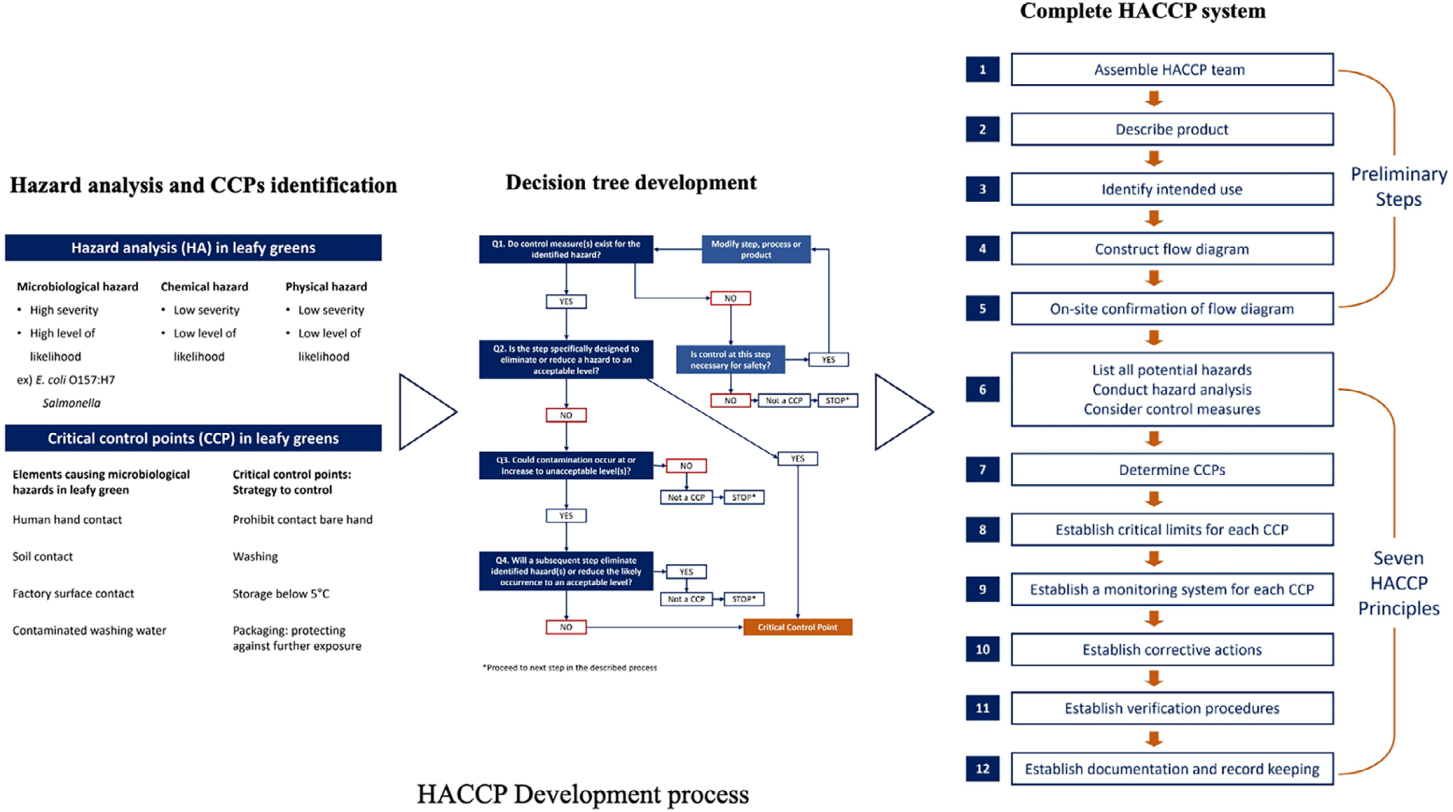
Process of HACCP development for leafy greens (constructed based on WHO 2008; FDA, USA 2022; USDA 2020).

**FIGURE 4:**
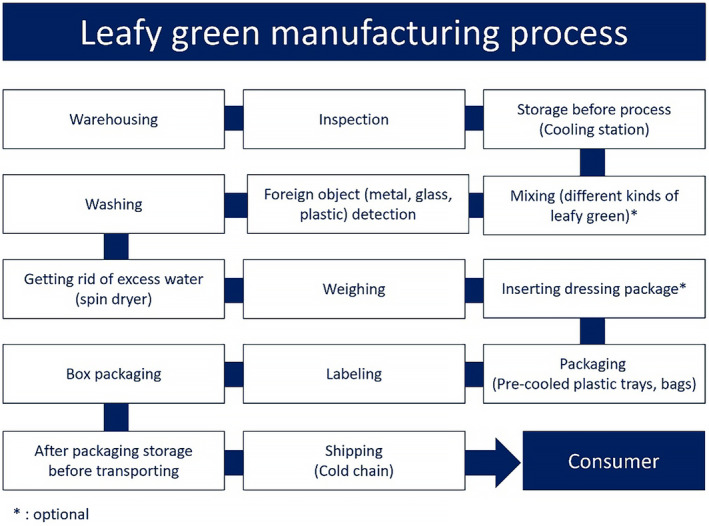
Process flowchart for the leafy greens (fabricated from processing vegetables of UC Agriculture and Natural Resources (2019)).

Leafy greens are typically harvested manually, and even when mechanical harvesting is used, there is still a risk of contamination during sorting, trimming, and packaging processes due to potential contact with hands or the environment (WHO 2008). Since disinfecting contaminated leafy greens is challenging without compromising product quality, Good Manufacturing Practices (GMPs) are essential. The development of an effective HACCP plan assumes proper management of leafy greens during cultivation. To address microbiological hazards, critical control points (CCP) for leafy greens may involve measures such as (1) avoiding contact with potentially contaminated surfaces like sinks by using colanders, (2) refraining from bare‐hand contact, and (3) maintaining storage temperatures below 5°C (South Carolina Department of Education 2016). A comprehensive HACCP development process (Figure 3) ensures the nutritional quality and safety of produced leafy greens based on the manufacturing process (Figure 4) within the leafy green industry.

## 5 Solutions to Prevent Preharvest Contamination

Preventing contamination in leafy greens involves controlling the quality of soil and irrigation water and closely monitoring interrelated factors such as soil amendments, irrigation methods, proximity to animal activity, and human hygiene practices. Adopting GAP standards allows farmers to take preventive measures to restrict pathogen entry significantly. The FDA, in collaboration with the EPA, launched the Leafy Greens STEC (Shiga toxin‐producing *E. coli*) Action Plan in 2020 as an extension of the FSMA Final Rule on Produce Safety (PSR), focusing on prevention, response, and addressing knowledge gaps (US FDA, Leafy Green STEC Action Plan 2020). This plan includes new regulations on antimicrobial agents for preharvest irrigation water, reports on investigated outbreak areas, and studies to understand pathogen survival in the environment. Table 3 lists the effective preventative measures for restricting pathogen entry into produce.

**TABLE 3:** Measures for preventing and controlling preharvest contamination of leafy greens (FSMA).

On‐farm factors	Challenges	Potential solutions/preventative measures (FSMA, GAP, and PSR)
Production field	If a production field is adjacent to livestock management on the same level, then cross‐contamination occurs. If the land was used previously for nonfood cultivation, then pathogens may be present. Flood zones may already have pathogens in the soil	Production fields must be located uphill from animal areas to prevent runoff from flooding Avoid using the fields previously used for animal activities or heavily sprayed with chemicals Avoid fields that are regularly flooded or susceptible to runoff
Manure/soil amendment	Utilization of improperly composted organic manure or animal materials (bone meal, feathers) as soil amendments creates a favorable environment for pathogens. Manure application close to the day of harvest may promote pathogenic transmission	Apply untreated manure 9 months before harvesting. Apply soil amendments and added chemicals long before harvesting, when the soil temperature is warm (> 50 °F), to avoid contamination Apply fully composted manure and ensure that it does not encounter produce/greens. For leafy greens, manure application must be applied before 120 days of harvesting. Manure must be stored in a clean environment away from the cultivation area
Hygienic practices	Absence of restrooms may promote additional possibility of human pathogen exposure. If fresh produce is touched with dirty hands, the produce can be contaminated. Sick workers can transmit disease‐causing pathogens to other workers and produce	Bathrooms must be constructed for the farm workers to prevent human pathogens or fecal presence around cultivation. Proper handwashing must be done prior to touching the produce. Workers must not work in production fields while sick. Boils, cuts, or wounds must also be properly secured to prevent contact between blood and the produce
Water for preharvest (agriculture water, wastewater, surface water, groundwater, municipal water, or groundwater)	Increased outbreaks related to (preharvest) agricultural water or water runoff from the adjacent land with produce farms (US FDA, Agricultural water use on produce 2021). Untreated wastewater poses high chemical, physical, and microbiological hazards. Surface water is more exposed to feces, pollutants, and harmful microbiota. Gray water such as municipal water may contain up to 5 log_10_ units of viral particles per liter (Wu et al. 2020). Groundwater stored in underground aquifers is low in pathogens and hazardous metals	Annual “agricultural water assessment” to monitor water quality for its potential hazards and introduce corrective and mitigating measures to minimize the potential of hazardous contamination. Microbial quality should be monitored and assessed regularly (three times annually). Water treatments, like filtration, coagulation, disinfection, flocculation, and irradiation, are desirable to cut down the pathogenic load, and special attention is required to eliminate the viral load from the recycled gray water before applying to leafy greens. The geometric mean (GM) of microbiological testing of water used during growing, harvesting or postharvesting activities should be 126 CFU or less for generic *E. coli* per 100 mL of water (Truitt et al. 2018). Groundwater is the safest water that can be utilized as it has a low pathogenic load. If surface water or effluent water is used, then it should be treated by filtration, coagulation, or flocculation. Ultraviolet radiation must be used to reduce biological hazards.
Irrigation method	Sprinkler methods have a high potential to contaminate leafy greens through the transmission of viral particles with aerosol or droplet formation during irrigation	Drip or subsurface irrigation must be used so that water does not encounter the edible portion of the produce
Animals	Animals can shed pathogens, such as *Campylobacter*, *Salmonella* spp., and *Listeria*, through fecal matter, saliva, or body contact	Tall fences should be installed around fields to restrict the passage of cattle in the harvesting area. Excess use of chemicals, like fertilizers and pesticides, must also be avoided

## 6 Conclusions

Leafy greens, often consumed raw, are consistently vulnerable to pathogen contamination. Preventing the proliferation of harmful pathogens during postharvest processing requires restricting pathogen internalization and biofilm formation at the preharvest stage. Controlling soil and water quality is crucial for maintaining the quality and safety of leafy greens. The adoption of FSMA and GAP measures and implication of HACCP enables handlers of fresh produce to reduce potential hazards, ensuring the nutritional quality and safety of leafy greens. Despite numerous precautions and measures, completely restricting pathogen entry into leafy greens is challenging. Rapid detection methods are available to identify the pathogen presence, potentially saving time and lives. Future studies on these methods are recommended for the quick identification of pathogens in leafy greens, which could prevent multistate outbreaks and protect consumer health.

## Author Contributions

**Ukti Bimal Sheth:** data curation (equal), formal analysis (equal), investigation (equal), writing – original draft (equal). **Md Ariful Haque:** data curation (equal), formal analysis (equal), investigation (equal), methodology (equal), visualization (lead), writing – original draft (lead), writing – review and editing (equal). **Min Ji Jang:** investigation (equal), writing – review and editing (equal). **Samuel Haruna:** data curation (supporting), investigation (supporting), software (equal), writing – review and editing (supporting). **Tony V. Johnston:** data curation (supporting), funding acquisition (supporting), writing – review and editing (supporting). **Deokyeong Choe:** formal analysis (supporting), investigation (supporting), writing – review and editing (supporting). **Ying Gao:** funding acquisition (supporting), investigation (supporting), writing – review and editing (supporting). **Seockmo Ku:** conceptualization (lead), data curation (supporting), formal analysis (supporting), funding acquisition (lead), investigation (lead), project administration (lead), resources (lead), supervision (lead), visualization (supporting), writing – review and editing (lead).

## Conflicts of Interest

The authors do not have any conflicts of interest. Ukti Bimal Sheth and Md Ariful Haque have contributed to building the concept, acquisition, analysis, and interpretation of data and writing the manuscript; Min Ji Jang contributed to the writing section; Samuel Haruna, Tony V. Johnston, Deokyeong Choe, and Ying Gao provided valuable suggestions and revised the manuscript, Seockmo Ku contributed to building the concept, guiding, providing suggestions, and revising the manuscript critically.

## Data Availability

The data that support the findings of this study are available on request from the corresponding author.
